# Captured voices in cancer: experiences from networking between individuals with experiential and professional knowledge

**DOI:** 10.1186/1472-6963-7-23

**Published:** 2007-02-19

**Authors:** Christina Carlsson, Kerstin Segesten, Mef Nilbert, Kerstin Nilsson

**Affiliations:** 1 Institute of Clinical Sciences, Department of Oncology, Lund University, Lund, Sweden; 2 Department of Health Sciences, University College of Borås, Borås, Sweden; 3 Faculty of Health and Caring Sciences, Institute of Nursing, The Sahlgrenska Academy at Göteborg University, Göteborg, Sweden

## Abstract

**Background:**

Patients needs and experiences attract increasing attention within health care. In order to generate knowledge about the voices that emerge from collaborative experiences between members of patient associations for cancer patients (PACP) and health care professionals (HCPs), we studied a permanent network aimed at improving cancer care through increased attention to the cancer patients' view and experiences.

**Methods:**

Open-ended interviews were carried out with 16 individuals; 6 PACP members and 10 HCPs, and after transcription the texts were analysed by inductive content analysis.

**Results:**

Four *voices*, which represent various experiences from networking, were identified; *the hesitant voice*, *the enlightened voice*, *the liberated voice*, *and the representative voice*. The hesitant voice reflects uncertainty experienced when the participants were exposed to different views and opinions within the network. The enlightened voice reflects new points of view and gain of knowledge. The liberated voice signifies trust, balance, and confidence related to individual experiences and responsibilities being viewed in a broader perspective. The representative voice is derived from the transformation of experiences and responsibilities through insight, understanding, and new perspectives.

**Conclusion:**

Networking between representatives for PACPs and HCPs may help the participants manage uncertainty, strengthen the patient's perspective and provide new views on common issues. The different voices identified in this study demonstrate that both PACP members and HCPs distanced themselves from their individual experiences in order to be perceived as unselfish and knowledgeable within the network. Although the climate was characterized by trustfulness, the members' unique positions need to be defined in order to obtain an optimal balance between the groups and prevent members' patient experiences of losing their character by learning to much from the HCPs. Increased understanding of the hesitant, the enlightened, the liberated, and the representative voices, and awareness of experiential versus professional knowledge of cancer may facilitate and probably improve future networking efforts.

## Background

Individuals with cancer enter the health care system as users bringing their life stories, based on personal experiences, including those from disease or illness [1]. Increasing demands are being made for health-care to become user-oriented with increased attention to patients' perspectives and needs – and thereby emphasize the individuals' life world experiences. Such perspectives may facilitate interaction between professional and experiential knowledge and thereby obtain balance between the life-world perspective carried by the individual and the medical perspective carried by health-care professionals [2-5]. Since cancer affects both physical and psychological well-being, most cancer patients require long time, sometimes several years, to cope with their new experiences and to learn to live with cancer [6-9]. Cancer patients have increasing possibilities for involvement in activities offered by patient associations for cancer patients (PACPs) [10]. PACPs in Sweden have various types of members; individuals affected by cancer, support members who may represent family members or health care professionals (HCPs). Swedish PACPs belong to civil society and have been described by the Nordic civil society researchers using the metaphors "voice" and "service" [11]. An interest in change and possibilities to influence health care are predominant reasons for involvement in terms of being a "voice", whereas common activities and meetings, support groups, and disease-related information represents "service" [10-12]. The patient's role within the health care system is changing with patient representatives increasingly taking part in health-related task forces, but research around their views on such involvements is scarce [13-17]. In order to generate knowledge about the voices that emerge from collaborative experiences between PACP members and HCPs, we studied a permanent network aimed at improving cancer care through increased attention to the cancer patients' views and experiences.

## Methods

### Participants and data collection

The study was performed within a network for patient representatives (PACP members) and HCPs that was initiated in project form with the aim to improve cancer care and its results have been presented elsewhere [18]. Meetings were held three to four times annually and took place within the health care facilities and during the HCPs' working hours. After three years the project was made permanent (by the participants) and remains with the same aim. In order to gain a broad picture of the v long-term experiences from networking the PACP members and HCPs who remained in the network after 5 years (8 PACP members and 19 HCPs) were eligible for the study. To keep balance between participants representing PACPs and HCPs, all 8 PACP members and the first 12 HCPs from an open network organisation list (not in alphabetical order) were selected for interviews. The interviewer (CC) contacted the prospective informants by telephone with information about the aim of the study and an invitation to participate. An introductory letter was then sent to the prospective informants that included the purpose of the study, a confidentiality agreement and information about voluntaries. Of the 20 individuals invited, two PACP members declined to participate because of work-related priorities and feelings of being a stand-in representative, respectively, and two HCPs declined to participate because of a lack of experience and time. Hence, the study was based on 16 individuals: six members and ten HCPs from hospital care, primary health care and community-based health care. Characteristics of the informants are presented in Table 1.

**Table 1:** Characteristics of the informants

	PACP members (*n *= 6) ^1,2^	HCPs (*n *= 10) ^3^
**Age**, mean (years)	64	50
Gender:		
Male	4	1
Female	2	9
**HCPs**:		
Years of working experience (mean)		19.5
		
**HCPs profession**:		
M.D.		2
R.N.		4
Physiotherapist		1
Priest		1
Secretary		1
Social worker		1
**PACP for**:		
Breast cancer	2	
Colo-rectal cancer	2	
Head and neck cancer	2	
**Years of membership in PACP **(mean)	13.5	

^1 ^Personal experience of cancer and cancer within the family (*n *= 4)

^2 ^Had a special assignment within the PACP (*n *= 4)

^3 ^Experience of cancer within the family (*n *= 7)

Tape-recorded, open-ended interviews were used for data collection [19]. The initial exhortation was "I would like you to describe five years' network experience between PACP members and HCPs as you see it..." The interviews with the PACP members took place in their homes and interviews with the HCPs were held in a room outside the medical facilities. Each interview lasted about 45 minutes and was transcribed verbatim. The study design was judged according to Swedish legislation not to need ethics review, but the study was performed according to ethical research principles [20].

### Data analysis

The transcribed texts were analysed by inductive content analysis [21,22]. The first author (CC, a nurse specialised in oncology) read and re-read the transcribed texts to become familiar with the data carefully focusing on the aim of the study. Words, statements and sentences corresponding to the aim of the study were noted and grouped into preliminary themes. The content of each theme was expanded or reduced and sub-themes were formed by reflecting and bringing new questions to the text, such as: what does this specific statement say about experience? [22]. In order to avoid influence from preconceptions about the field, co-analysers with different experiences were used; KN, with experience in nursing and qualitative analysis, KS, with experience of qualitative analysis and of research in illness and care, and MN, with experience in oncology. The first author's analysis was used as the starting point for KN who examined the primary coded text, followed by KS who analyzed the consistency in the data analysis and MN participating in discussions. Here after consensus on the final themes and sub-themes was reached. In this process the authors reflected on subjectivity and preconditions related to the participants' different viewpoints. To illustrate the content of the themes and sub-themes we use excerpts that refer to the identity (PACP member/HCPs) of the individual participants' statements.

## Results

Four *voices*, which represent various experiences from networking, were identified; *the hesitant voice*, *the enlightened voice*, *the liberated voice*, *and the representative voice *(table 2). The *hesitant voice *reflects uncertainty when the participants experiencing insecurity and resistance. The *enlightened voice *reflects listening to new points of view and gaining of knowledge. The *liberated voice *signifies trust, balance, and confidence related to putting individual experiences and responsibilities into a broader perspective. The *representative voice *is derived from the transformation of experiences and responsibilities through insight, understanding, and new perspectives.

**Table 2:** Themes and sub-themes

*Themes*	*Sub-themes*
**1. The hesitant voice**	1:1. Uncertainty
	1:2. Insecurity
	1:3. Coping

**2. The enlightened voice**	2:1. Accessibility
	2:2. Learning
	2:3. New points of view

**3. The liberated voice**	3:1. Reflections
	3:2. Trustfulness
	3:3. Balance

**4. The representative voice**	4:1. Transformation
	4:2. Influence

### The hesitant voice

#### Uncertainty

Uncertainty arose when PACP members and HCPs faced each other's experiences and differences in opinions within the network e.g. in the HCPs' reactions to how the PACP members introduced themselves. One PACP member felt that professionals reacted negatively in a discussion around heredity:

/.../Once when I was going to present myself [at the network] I talked about heredity...and then it was like I said too much/.../Yes, I felt it/.../(PACP member 3)

The PACP felt that the HCPs were uncertain about how to interpret feedback and speculated about whether this could be caused by current trends in health care in which patients' experiences cannot be ignored. The PACP members also felt uncertain about the significance of their own personal experiences and functions in the network; can individual experiences make a difference when the person perceives that he/she had little to add to a conversation or when his/her opinion was not based on his or her own cancer experience?

The HCPs, on the other hand, described uncertainty about how they should handle the experiences presented by the PACP members and what position they should take in this new relation. These feelings were further strengthened in the HCPs' relations with two PACP members with professional experience of health care (one nurse and one social worker). Were they describing their own or other members' experiences, or were their experiences derived from work within the health care sector? HCPs also felt uncertain when members discussed personal experiences in the network. The HCPs wanted to differentiate between general and individual problems/experiences:

I [HCP] think sometimes that it is difficult in such broad contexts when a patient [PACP member] talks personally about himself or herself/.../when you belong to a large network, one that works on another level, making things good for patients, that comes out, that personal experience (HCP 8).

#### Insecurity

Insecurity arose when PACP members and HCPs experienced resistance, differences in opinion, and criticism within the network. PACP members described how they had to argue in favour of the patients' perspective when they confronted differences in interpretation in opinion:

Then suddenly it's interpreted [HCP's interpretation] like ' you mean that's how you felt'. I didn't mean that. I mean how it feels to be a patient/.../(PACP member 8)

Similarly, HCPs felt criticized, although not personally, and stated that they felt a sharp tone in the criticism:

/.../he makes comments all the time. Of course, he has a sharp profile, too, a person who has opinions about things/.../(HCP 1)

Both PACP members and HCPs described how they gradually perceived the shortcomings and limitations of the network. The PACP members described gaining knowledge through the network but reported that this had not always been beneficial since such new information could be frightening:

/.../It can be a little frightening sometimes, too, of course. Once when he [the doctor] talked about genes...it was pretty awful actually/.../(PACP member 4)

#### Coping

PACP members described how they handled feelings of uncertainty in the network by forming an idea about the HCPs' opinions and professional arena in order to judge and determine which experiences could be presented. The members thus took a watchful and rethinking attitude for example: (yes, you see a lot in the body language and what kind of person [the HCP] it is I'm going to talk to now (PACP members 1 and 5, respectively) and some times waited for an opportunity to get their ideas across to the HCPs at a later occasion.

When HCPs were confronted with criticism from the PACP members, they first tried to determine whether the criticism was based on personal disappointment in the care given which sometimes turned out to be the case.

/.../who was maybe disappointed at times and we've heard things [lately in the network] like that (HCP 1)

This approach demonstrates that the HCPs also apply coping strategies to adapt to the relations in the network.

### The enlightened voice

#### Accessibility

The PACP members described how communication within the network strengthened the patient's voice as discussions often started from their perspectives:

/.../Since the patient associations, the patients' voices were a part of things from the beginning/.../so things often started from the viewpoint of our opinions/.../since we [PACP members and HCPs] got on that track [the patients'] from the beginning, it was easier to stay there/.../(PACP member 2)

The network sanctioned HCPs to reach out to PACP members with information and to access patients' experiences:

/.../they [PACP members] have described/.../they [PACP members] talked about their experiences, not from the perspective of health care but from their [patient] perspective then, that they were sick (HCP 5)

HCPs also describe how PACP members presented their experiences with empathy, e.g. when PACP members answered HCPs' questions, shared their views on certain subjects or made a contribution at a network meeting. In conversation, PACP members based their statements on what was perceived as important, either from their own or from other PACP members' point of view and considering the basic principles of their association:

Well, I've tried to share my experiences and what the Association thinks, You have to think about what you want to have said and what you want to help with and things like that (PACP member 5)

Hence, the network developed into a forum for expression and validation of experiences, be they personal or related by others.

#### Learning

In the network, the participants had the opportunity to learn about e.g. cancer care, patient associations, and the patient's perspective. When PACP members could talk together and reflect upon care and medical situations, they often reported that they gained insight into how certain situations developed:

It was this business with [name of the medication], I went for a whole year and felt freezing (PACP member 7)

I've learned many, many things/.../I've also gotten explanations about things I didn't know about before/.../so I've learned a lot about health care/.../I understand why they, why people [health care] do things in a certain way and why it [the care situation] is the way it is (PACP member 2)

HCPs reported increased knowledge about different cancer types and developed an awareness of common phenomena such as crisis reactions and coping. The HCPs stated that their understanding of patients' experiences and perspectives had changed, especially concerning patients' needs and rights, reaching joint decisions and applying the user's involvement in care. HCPs reported that the scope and the variety of PACP members' expressions of experiences helped them gain an understanding of which issues were perceived as relevant and to focus on these:

We [PACP members and HCPs] had one of those meetings [a network meeting] and then one [PACP member] talked a lot about how it is to be sick. And it helped me a lot to understand their situation, how things had been (HCP 5)

HCPs described that they gained knowledge valuable for their professional development and everyday work:

/.../and it [the network and its health care consumers] I think has enriched my job and my development an incredible amount during these years (HCP 7)

As the PACP members represented their associations in the network, the HCPs also received descriptions of the associations' goals and activities as well as how PACPs see themselves as being a complement to the health care system. This demonstrates that the network developed into an arena where knowledge was generated and communicated from health care workers and volunteers.

#### New points of view

HCPs described that the network experiences influenced their views on patients' needs. In particular, the belief that patients want **as **much contact as possible with the health care system was not supported. Another issue that was re-evaluated had to do with the view that HCPs can improve patients' issues without taking patients' opinions into consideration:

I think we're [HCPs] bad at that in general, [HCPs] believe that we work in a good way but we don't try to get in the patients' opinions, but instead we spin it together ourselves.../.../(HCP 9)

The value of patients' experiences was also reflected in the HCPs mentioning that it is anticipated that future consumers of health care will have a greater awareness of the patient's perspective and needs, and that this will require close collaboration between health care and patient representatives. It also became evident that, although the HCPs were experts in cancer care, they lacked personal experience:

/.../they [PACP members] really have more experience by really having had the disease as opposed to the care personnel/.../I think that's a huge difference (HCP/)

### The liberated voice

#### Reflections

The HCPs stated that it was easier to be open-minded and receptive to the experiences described by PACP members from their own area in cancer care. PACP members also expressed how interest varied between professionals and was related to their experiences:

But I [PACP member with a background as an RN] actually have a feeling that people have listened to me more than it would have been if it were someone from another patient association (PACP member 1)

Thereby, HCPs tended to pay more attention to the views presented by certain PACP members. HCPs also mentioned that it required courage to discuss disease-related issues with individuals with patient experience outside of the health care system. The HCPs reflected upon whether they were unwilling to relinquish power, on how close they accepted becoming involved with the PACP members' experience, and on the limits of their ability to influence:

One [HCPs] can see it as a threat, that one perhaps doesn't want the patient to come too close and gain too much influence (HCP 7)

HCPs had been confronted with their own doubtful attitude toward patient associations and with the other HCPs' negative attitudes toward patient associations. Awareness of PACPs position that affected the PACP members' freedom to speak and that made them feel inferior is illustrated in the following:

I think maybe that they [PACP members] can feel that they are a little bit in a weak position when we talk our language [the language of professionals] (HCP 9)

The HCPs also questioned whether the PACP members were able to represent more general patient perspectives and experiences.

#### Trustfulness

The confident environment has to do with PACPs members' courage and honesty in the conversation. When the PACP members felt that the HCPs listened, their courage and willingness to provide further details grew:

/.../You [PACP members] dared to be open in another way and if you feel that they [HCPs] listened, were interested and have a positive attitude, then I [as PACP member] gain courage to bring up things that I wouldn't otherwise have brought up/.../(PACP member 2)

There can be a risk that those HCPs, as providers, may suppress the PACPs into silence. In that scenario the HCPs spoke with admiration about the PACP members' courage in talking about their experiences and presenting their views:

In spite of the fact that there are professionals here, both doctors and nurses, they [PACP members] have the courage to say something, they are brave enough to protest when they think that something is wrong, they're not afraid of that (HCP 7)

The HCPs became more aware of how patients' representatives had a tendency to be quiet, to keep a low profile and to give critique unwillingly. In contrast, the HCPs were outside their role as providers of health care and this independence allowed honesty in the conversations within the network.

#### Balance

A balancing act was evident in the sense that PACP members protected their unique personal experiences from, cancer, but at the same time distanced themselves from their own experiences when they represented broader views. PACP members considered it important to distance them from the HCPs' professional experience:

/.../you [as PACP members] can't learn too much, you can't go so far that you start to see it from the [health care's] perspective/.../(PACP member 2)

In a similar way the HCPs described how they balanced the different perspectives through distancing themselves from their professional responsibilities in order to regard the other network participants as representatives rather than as patients. The difficulties when patient talk about herself personally can be exemplified in a quotation from a HCP:

Yes, it's been both difficult and good/.../I sometimes think that it's hard in such a large context [the network] when a patient talks about herself [personally] (HCP 8)

Thus, both PACP members and HCPs distanced themselves with respect to their different and unique experiences in order to be perceived as unselfish and knowledgeable, and as representatives of comprehensive questions and common interests.

### The representative voice

#### Transformation

The PACP members had to transform their own feelings as former patients in order to understand and handle other individuals' experiences. At the same time that they exchanged experiences with representatives of the health care and medicine they had to tackle their own ambivalence about "learning too much":

/.../you [as PACP member] can't learn too much, you can't go so far that you start to see it from that [health care] perspective (PACP member 2)

In a similar way the HCPs had to make transformation, i.e. maintain a distance to their professional responsibilities:

#### Influence

The network contributed to greater insight into the different realities and perspectives that the participants represent. PACP members could develop broader insight into the health care system and could thereby examine and understand the flow of information, provide feedback to their associations, and, through applying the patients' perspective, could directly give suggestions that may perceived useful by the HCPs. Applying patients' perspective and the direct way to do this in the network could be exemplified in the following quotations:

So you've been able to opinions sometimes, how it is to be, in part to have gone through a cancer illness and in part I've had surgery (PACP member 5)

I told them [HCPs] that it in fact can be that way too (PACP member 2)

Within the network HCPs often contributed with novel ideas and took responsibility for progress and reaching the goals agreed on. The HCPs also reported how innovative ideas had been conveyed and how they felt that the experiences from the network had helped them focus on the patient's perspective:

Both HCPs and PACP members described that changes had been made because of the collaboration. Two examples of these changes, illustrated in the following quote, is the so-called "open returns", which implies that the patient has the right to directly contact or come to the ward at which previous treatment and care was given and that changes in thinking:

/.../I've felt that he [the doctor] and many others have changed their minds when I started to talk about "open returns"/.../(PACP member 1)

/.../She [the nurse] says that she remembers that and that she thinks about it...so that in some way I guess maybe it's started o take a root a little (PACP member 2)

## Discussion

Patients' needs and right to information and involvement in health-care are increasingly recognized and constitute the basis for this study on experiences from networking in cancer care [23]. Through interviews with PACPs and HCPs with five years of networking experiences we identified different voices reflecting experiences from interaction and communication within the network; *the hesitant, the enlightened, the liberated*, and *the representative voice*. Concordance between the analysers can be seen as strength but as the same time may disguise important differences. For that reason we tried to carefully focus on the aim and how the interpretation should be understood in the context (networking) of experiences and voices from different cultures. Overall, networking provided possibilities for meetings between PACP representatives and HCPs outside of their traditional roles as patients and caregivers, respectively, and this opportunity brought about reflections on their different responsibilities and promoted views from the cancer patient's life-world. These findings are in line with the voice theory, in which the "voice of life-world" represents natural attitudes and experiences in everyday life, whereas the "voice of medicine" represents the technical possibilities and scientific knowledge in health care [24]. The latter has by tradition been the predominant in health care, but the knowledge generated from networking led to increased insight into the patient's perspectives and experiences. The HCPs acknowledged the importance of developing a common knowledge basis that encompassed both perspectives and reflections contributed to an increased awareness of the imbalances between the "voice of the life world" and the "voice of medicine". Within patient-focused communication, joint engagement in the "voice of the life world" by both patients and health care personnel is referred to as the 'mutual life world' and according to Barry [25] has been reported to improve communication about both of physical and psychological symptoms.

HCPs reported that the networking experiences positively affected their professional practice and strengthened the patient perspective, which suggests that visualization of the patient's perspective, e.g. in joint activities and collaborative projects, may ease interaction and reduce uncertainty. Sätterlund Larsson [2,26] stated that, although the "voice of medicine" has dominated health care, both voices are recognized to be needed, and Sarangi [2,26] related such domination to the facts of institutional order. However, involvement needs to be accepted within health care, should optimally involve different health care areas and HCPs representing different areas of expertise, and include also information and education on e.g. care programs, treatment guidelines, and clinical research [16,27,28]. Patients' needs and rights can exemplify awareness, and the experiences gained within the network suggest that the HCPs were insufficiently aware hereof. The National Swedish Board of Health and Welfare have issued demands for health care to be user-oriented and to give more attention to the "voice of life world" [29, 30]. Uncertainty in the network interplay was expressed as *the hesitant voice*, but reflection and self-criticism were also facilitated and allowed expression of *the liberated voice*. The reflection and self-criticism provided a possibility to compare different experiences. Bookman [4] talked in that sense about experiential knowledge in voluntary common. These reactions underline the importance of collaboration, particularly against the background of the different interpretations of emotional reactions. These differences concerning personal and psychological aspects, and quality of life versus medical information, are known from other studies [31-34]. However, despite anticipated difficulties, individuals with different perspectives and backgrounds may have difficulties in engaging in discussions and considering collaborative issues. The PACP members' possibilities to listen and exchange experiences have been studied [10] and, in our findings, *the enlightened voice*, is transformed into *the representative voice *in discussions with HCPs. Several PACP members reported an ambivalence in 'learning too much', with a sense of moving away from the patient's experiences of the disease to a representative voice carrying extensive knowledge about cancer. This is especially evident when participation in the network requires "professional" patient representatives, such as the PACP's contact persons who have been trained in facts and treatment about cancer as well as in psychological reactions, and who themselves receive continuous support in their meetings with other individuals with breast cancer (the most famous being the Reach-to-Recovery Program) [35].

Can extended networks have an impact on cancer care? For cancer patients the network provides information about other patients' experiences and a higher awareness of both the strengths and the weaknesses in health care. The study also demonstrates how greater emphasis on the user's/consumer's perspective must be based on respect and understanding, demonstrated through contributions from the different voices identified.

## Conclusion

Networking between representatives for PACP and HCPs may help the participants manage uncertainty, strengthen the patient's perspective and provide new views on common issues. The different voices identified in this study demonstrate that both PACP members and HCPs could distance themselves from their individual experiences in order to be perceived as unselfish and knowledgeable within the network. Although the climate was characterized by trustfulness, the members' unique positions to present patients' experiences need to be defined in order to obtain an optimal balance between the two groups and prevent members' patient experiences of losing their character by learning to much from the HCPs. Increased understanding of *the hesitant*, *the enlightened*, *the liberated and the representative voices*, and awareness of experiential *versus *professional knowledge of cancer may facilitate and probably improve future networking efforts.

## Competing interests

The author(s) declare that they have no competing interests.

## Authors' contributions

CC: Study design, data collection, data analysis and interpretation of data, writing the manuscript and responsible for the financial issues of the study.

KS: Contributed critical revision: of the consistence in the analysis and interpretation of data.

MN: Participating in discussions and writing the manuscript

KN: Study design, data analysis (examined the pre-coded text) and interpretation of data, and writing the manuscript.

All authors have read and approved the final manuscript.

## Pre-publication history

The pre-publication history for this paper can be accessed here:


